# Stable isotope data and radiocarbon dates from Brazilian bioarchaeological samples: An extensive compilation

**DOI:** 10.1016/j.dib.2022.108117

**Published:** 2022-04-03

**Authors:** Caroline Borges, Ingrid Chanca, Kevin Salesse

**Affiliations:** a Department of History, Federal Rural University of Pernambuco, Dom Manuel de Medeiros Street, s/n, 52171-900 Recife, PE, Brazil; b Laboratório de Radiocarbono, Instituto de Física, Universidade Federal Fluminense, Av. Litorânea s/n, 24210-346 Niterói, RJ, Brazil; c Department of Biogeochemical Processes, Max Planck Institute for Biogeochemistry, Hans-Knoell-Straße 10, 07745 Jena, Germany; d Department of Anthropology, Faculty of Science, Masaryk University, Kotlářská 2, 611 37 Brno, Czech Republic

**Keywords:** Carbon, Nitrogen, Oxygen, Strontium, Radiocarbon dating, Collagen, Bioapatite, Stable isotope analysis, Archaeology, Brazil

## Abstract

Three decades have passed since the publication in 1991 of the first use of stable isotope analysis applied to a Brazilian archaeological context. Despite being still mainly applied to palaeodietary research, stable isotope analysis in archaeology has been diversified in Brazil. In the last five years, an increasing number of studies has addressed various issues. Such issues are related to population mobility, social differentiation, health and children care, changes and resilience of cultural practices, and identification of the origin of enslaved populations brought by force from the African continent, among others. However, research in this area is still incipient when compared to the large territory of Brazil (WGS 84: -33˚ to 5°N, -73˚ to -34˚E), the diversity of socio-cultural contexts of pre-colonial and indigenous societies, and the country's historical formation process. In terms of radiocarbon dates, data are also sparse and lack essential information as the material used for dating, as this information could be related to necessary corrections, e.g., the marine reservoir effect. The first radiocarbon dates of Brazilian archaeological material are reported, however, since the 1950s and have been more frequently reported in publications across Brazil since the installation of the first Brazilian radiocarbon laboratory (CENA/USP) in 1990 and the first Latin American ^14^C-AMS facility (LAC-UFF) in 2012. Thus, the purpose of this compilation was to gather all dispersed, and often fragmented, data from analyses of stable and radioactive (focusing on radiocarbon) isotopes carried out in Brazilian archaeological contexts. We compiled data from 1991 until the end of November 2021. The data included here contain information from 71 archaeological sites, 556 humans, 219 animals and 2 plants. Isotopic analyses were performed on 832 organic samples, mainly paired δ^13^C and δ^15^N plus δ^34^S measurements, and on 265 mineral samples, mainly δ^13^C, δ^18^O and ^86^Sr/^87^Sr measurements. Sr concentrations for 49 mineral samples were also compiled. Radiocarbon or relative dates span from 18 kyr BP to the present. All data from this compilation are deposited in open access on the IsoArcH platform (https://doi.isoarch.eu/doi/2021.005). This extensive work aims to point out the gaps in stable isotopes and radiocarbon dates provided for Brazilian archaeological contexts that could be further explored. Besides, it aims to promote easy access to numerous analyses that, otherwise, would be hard to obtain. Lastly, it seeks to broaden the interdisciplinary collaboration in Brazil and strengthen the international collaboration among peers.

## Specifications Table

**Table:** 

Subject	Archaeology
Specific subject area	Bioarchaeology Biomolecular archaeology Stable isotope analysis Radiocarbon dating
Type of data	Table 1 – Summary of all the sites compiled in this dataset including IsoArcH Platform ID, names and references of the primary data (in brackets), Geographic localization with the Brazilian State and coordinates (latitudes and longitudes in WGS 84), Relative Age with the lower and the upper limits and age system, and number of samples (Human, Animal, Plant). Site ID corresponds to number shown on the map of Figure 1. Figure 1 – Map showing all archaeological sites included in this dataset. A key to the sites IDs is provided in Table 1 as Site ID.
How data were acquired	Data were collected from international, regional, and local journal articles, book chapters, research reports, master dissertations, and doctoral thesis, released between 1991 and the end of November 2021. Access to the literature was primarily through digital repositories. The language of most of the studies is Brazilian Portuguese.]
Data format	Raw
Parameters for data collection	This compilation includes all data that are reported with the individual references and isotopic and/or radioactive values of each sample.
Description of data collection	A systematic literature review was conducted using Google Scholar, the Brazilian repository of scientific articles Scielo (https://www.scielo.br/), the Brazilian repository of researchers CV *Plataforma Lattes* (https://lattes.cnpq.br/), and each different repository of masters dissertations and doctoral thesis of each Brazilian university with graduate programs in archaeology. In addition, we conducted personal communication with researchers and university library staff asking for works deposited only in their personal and/or institutional libraries, obtaining manuscripts inaccessible otherwise. Works published until 24 November 2021 are included in this compilation.
Data source location	This dataset contains information on stable isotope analyses and/or radiocarbon dates from 71 archaeological sites, 777 individuals, 556 humans, 219 animals and 2 plants; data contain 1097 isotope analyses, 832 were organic samples, mainly C, N and S measurements, and for 265 mineral samples, mainly C, O and Sr measurements, from present day Brazil (WGS 84: -33˚ to 5°N, -73˚ to -34˚E). Samples have radiocarbon or relative dates showing a chronology between 18 kyr BP and nowadays.
Data accessibility	Repository: IsoArcH (https://isoarch.eu/) [1] DOI of the dataset: 10.48530/isoarch.2021.005 Direct URL of the dataset: https://doi.org/10.48530/isoarch.2021.005 Data is available under the Creative Commons BY-NC-SA 4.0 license.

## Value of the Data

• Until the present date, no one has done a compilation of these stable isotopes and radioactive analyses in the context of Brazilian archaeology. We compiled data from 1991 until the end of November 2021.
• The purpose of this compilation was to gather all dispersed, and often fragmented, data from analyses of stable and radioactive (focusing on radiocarbon) isotopes carried out in Brazilian archaeological contexts
• The present compilation brings together all data on stable isotopes and radiocarbon dates on bioarchaeological samples scattered in the country published in different supports
• Most of the compiled data in this dataset are difficult to be accessed because they have not been published in peer-review journals, as they comprise master dissertations, doctoral thesis and research reports. When published in articles, these data appear in local journals, often limiting the reading by non-Brazilian Portuguese readers.
• This dataset will turn these data more accessible to the academic community. By this compilation, the scientific community will get easier access to stable and radioactive isotope data on bioarchaeological samples from Brazil.

## 1 Data Description

**Fig. 1:**
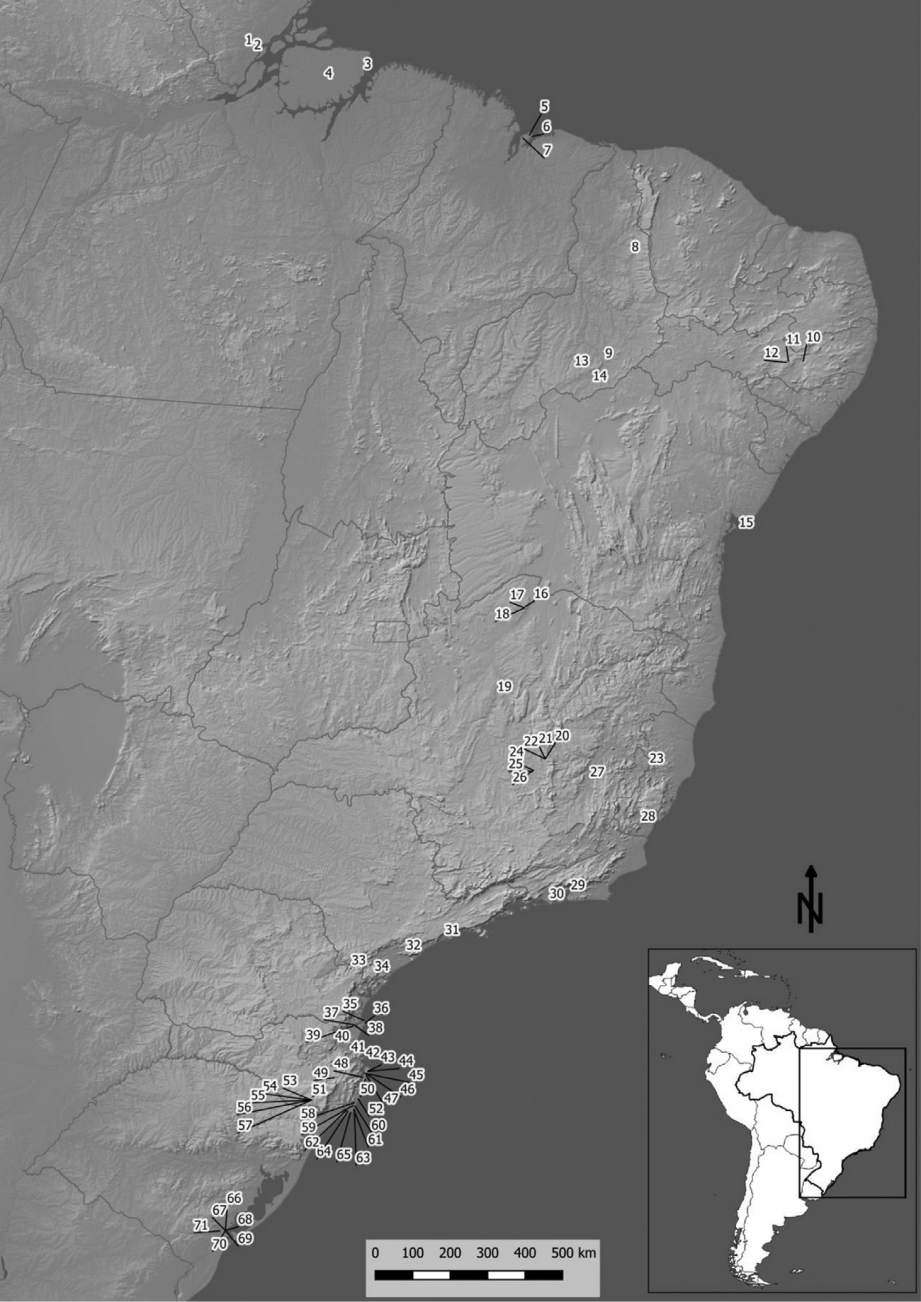
– Map showing all archaeological sites included in this dataset. A key to the sites IDs is provided in Table 1 as Site ID.

The dataset includes data from 71 archaeological sites, 777 individuals, 556 humans, 219 animals and 2 plants from all over Brazil (Fig. 1 and Table 1). In total, isotopic analyses are reported for 832 organic samples, mainly paired δ^13^C and δ^15^N plus δ^34^S measurements, and for 265 mineral samples, mainly δ^13^C, δ^18^O and ^86^Sr/^87^Sr measurements. Collagen quality criteria (collagen yield, carbon and nitrogen contents, and atomic carbon-to-nitrogen ratio) were included when available. Sr concentrations for 49 mineral samples were also compiled when reported. Samples date from 18 kyr BP to nowadays. Data are made available in IsoArcH (https://doi.isoarch.eu/doi/2021.005.), an open-access and collaborative isotope database for bioarchaeological samples (https://isoarch.eu/) [1].

**Table 1:** Summary of all the sites compiled in this dataset including IsoArcH Platform ID, names and references of the primary data (in brackets), Geographic localization with the Brazilian State and coordinates (latitudes and longitudes in WGS 84), Relative Age with the lower and the upper limits and age system, and number of samples (Human, Animal, Plant). Site ID corresponds to number shonw on the map of Figure 1.

						Relative Age			Total number of analyzed specimens		
IsoArcH platform ID	Site ID	Site Name [Reference]	Brazilian State	Latitude (WGS 84)	Longitude (WGS 84)	Lower Limit	Upper Limit	Age System	Human	Animal	Plant
1	50	Armação do Sul [4], [5], [6]	Santa Catarina	–27.75089	–48.502028	3065	1275	cal BP	48	18	
2	34	Capelinha 1 [7,8]	São Paulo	–24.83575	–48.243917	10180	6850	cal BP	1		
3	66	Pontal da Barra - PSG-07 [9], [10], [11]	Rio Grande do Sul	–31.77954	–52.234708	2027	1016	cal BP	1	10	
4	35	Forte Marechal Luz [12], [13], [14], [15]	Santa Catarina	–26.16642	–48.529331	1178	739	cal BP	32	5	
5	48	Praia da Tapera [5,6,14,15]	Santa Catarina	–27.68625	–48.571667	1280	935	cal BP	44	9	
6	32	Moraes [7,16]	São Paulo	–24.27407	–47.394661	6775	4887	cal BP	21	17	
7	31	Piaçaguera [16]	São Paulo	–23.86211	–46.3622	5894	5314	cal BP	13	3	
8	64	Jabuticabeira II [16], [17], [18], [19]	Santa Catarina	–28.59028	–48.960083	3137	1524	cal BP	79	7	
9	63	Galheta IV [16]	Santa Catarina	–28.56649	–48.788581	2010	2014	BC/AD	7	13	
10	43	Porto Rio Vermelho I - SC-PRV-01 [4,5]	Santa Catarina	–27.52283	–48.423078	4950	3652	cal BP	1		
11	47	Canto da Lagoa I - SC-CL-01 [4,5]	Santa Catarina	–27.61189	–48.479757	1535	1140	cal BP	1		
12	44	Porto do Rio Vermelho II - SC-PRV-02 [4,5]	Santa Catarina	–27.5234	–48.423717	1826	994	cal BP	16	18	2
13	49	Alfredo Wager - Alto Jararaca [4,5]	Santa Catarina	–27.69597	–49.332072	1700	1550	cal BP	1		
14	40	Ribeirão da Herta - SC-VI-16 [4,5]	Santa Catarina	–26.70293	–49.302214	1310	1280	cal BP	1		
15	53	São Joaquim - SC-RA-01 [4,5]	Santa Catarina	–28.29249	–49.937575	1270	1169	cal BP	1		
16	54	São Joaquim - SC-RA-03 [4,5]	Santa Catarina	–28.29249	–49.937575	1268	1159	cal BP	1		
17	55	São Joaquim - SJ-04 [4,5]	Santa Catarina	–28.29249	–49.937575	1305	1157	cal BP	1		
18	56	São Joaquim - SC-RA-05 [4,5]	Santa Catarina	–28.29249	–49.937575	1305	1270	cal BP	1		
19	57	São Joaquim - SC-RA-06 [4,5]	Santa Catarina	–28.29249	–49.937575	1267	1157	cal BP	1		
20	51	Urubici [4,5]	Santa Catarina	–28.00713	–49.589467	1830	1710	cal BP	1		
21	60	Caieira [5,18]	Santa Catarina	–28.4501	–48.771661	3731	515	cal BP	10		
22	61	Carniça [5,18]	Santa Catarina	–28.53999	–48.810836	3971	2153	cal BP	7		
23	62	Congonhas [5,18]	Santa Catarina	–28.56612	–49.000269	3727	3179	cal BP	9		
24	52	Imbituba - Vila Nova [5]	Santa Catarina	–28.26242	–48.690056	8000	1000	cal BP	1		
25	65	Garopaba [5]	Santa Catarina	–28.62514	–48.892356	8000	1000	cal BP	1		
26	58	Imaruí [5]	Santa Catarina	–28.363	–48.793667	8000	1000	cal BP	1		
27	46	Pontal das Almas [5]	Santa Catarina	–27.59539	–48.459222	8000	1000	cal BP	15		
28	45	Rio Lessa [5]	Santa Catarina	–27.57742	–48.519167	8000	1000	cal BP	4		
29	42	Porto Belo - Praia do Embrulho [5]	Santa Catarina	–27.14675	–48.481417	8000	1000	cal BP	1		
30	41	Laranjeiras [5]	Santa Catarina	–26.99722	–48.590861	8000	1000	cal BP	1		
31	39	Morro do Ouro [5,20]	Santa Catarina	–26.31463	–48.828	4824	4101	cal BP	26		
32	36	Enseada [5]	Santa Catarina	–26.23331	–48.498972	4514	1178	cal BP	17		
33	38	Espinheiro [5,21]	Santa Catarina	–26.28761	–48.798611	3337	933	cal BP	4		
34	59	Cabeçuda [18,22]	Santa Catarina	–28.4395	–48.82975	5079	3972	cal BP	5		
35	29	Sernambetiba [18,23]	Rio de Janeiro	–22.66639	–43.004139	1571	1748	cal BP	6		
36	37	Rio Comprido [20]	Santa Catarina	–26.27529	–48.807511	3835	3640	cal BP	16		
37	25	Lapa do Santo [24,25]	Minas Gerais	–19.50405	–44.03045	9889	9540	cal BP	25	8	
38	26	Lapa das Boleiras [24]	Minas Gerais	–19.52056	–44.068228	9540	9030	cal BP	2		
39	22	Santana do Riacho [24]	Minas Gerais	–19.17381	–43.694306	10000	1	cal BP	1	18	
40	24	Cuvieri [24]	Minas Gerais	–19.47849	–44.010025	6186	1	cal BP		18	
41	16	Lapa do Boquete [24]	Minas Gerais	–15.10219	–44.223167	8000	500	cal BP	6	8	
42	18	Abrigo Malhador [24]	Minas Gerais	–15.13814	–44.253667	898	676	cal BP	5		
43	17	Lapa dos Bichos [24]	Minas Gerais	–15.13396	–44.247711	11650	1	cal BP		21	
44	1	Gruta das Caretas [26]	Amapá	–0.076103	–51.511506	600	500	cal BP	15		
45	2	Gruta do Pocinho [26]	Amapá	–0.209444	–51.4686	600	500	cal BP	2		
46	3	Marajoara complex [2,26]	Pará	–0.716667	–48.523333	1600	500	cal BP	8		
47	33	Estreito [7]	Paraná	–24.67425	–48.856628	4011	3893	cal BP	2	1	
48	30	Pretos Novos cemetery [14,27,28]	Rio de Janeiro	–22.896	–43.192944	1769	1830	BC/AD	30		
49	15	Catedral da Sé de Salvador – Churchyard [14,28]	Bahia	–12.97328	–38.511806	1550	1856	BC/AD	12		
50	4	Marajó [2,26]	Pará	–0.966667	–49.566667	400	1300	BC/AD		2	
51	7	Bacanga [29,30]	Maranhão	–2.57877	–44.2818934	1840	1700	cal BP	1		
52	5	Paço do Lumiar [29,30]	Maranhão	–2.493647	–44.1107695	1190	990	cal BP	1		
53	6	Panaquatira [29,30]	Maranhão	–2.530786	–44.0418751	1800	1000	cal BP	3	23	
54	67	Pontal da Barra - PSG-01 [10,11]	Rio Grande do Sul	–31.77954	–52.234708	1790	1346	cal BP	3		
55	68	Pontal da Barra - PSG-02 [10,11]	Rio Grande do Sul	–31.77954	–52.234708	1816	1013	cal BP	5	8	
56	69	Pontal da Barra - PSG-03 [10,11]	Rio Grande do Sul	–31.77954	–52.234708	1403	1186	cal BP	1		
57	70	Pontal da Barra - PSG-06 [10,11]	Rio Grande do Sul	–31.77954	–52.234708	1575	1084	cal BP	3		
58	71	Lagoa do Fragata - PSGLF-02 [10,11]	Rio Grande do Sul	–31.79306	–52.3855278	2027	1013	cal BP	7	9	
59	12	Pedra do Cachorro [31], [32], [33], [34]	Pernambuco	–8.575423	–37.2467146	3967	566	cal BP	3	3	
60	10	Pedra do Tubarão - Cemitério do Caboclo [31]	Pernambuco	–8.539167	–36.8022222	1054	836	cal BP	3		
61	11	Alcobaça [31]	Pernambuco	–8.54	–37.1941667	958	798	cal BP	1		
62	9	Toca da Baixa dos Caboclos [3,[35], [36], [37]]	Piauí	–8.45	–42.0841667	524	1	cal BP	4		
63	14	Toca do Serrote do Tenente Luis [35], [36], [37]	Piauí	–8.814405	–42.4200562	915	304	cal BP	2		
64	13	Toca do Gongo I [35], [36], [37]	Piauí	–8.653291	–42.5303727	2000	2000	BP	2		
65	19	Buritizeiros [38,39]	Minas Gerais	–17.35885	–44.9656645	7000	6000	BP	1		
66	20	Santana do Riacho I [38,40]	Minas Gerais	–19.16859	–43.6920965	10000	8000	BP	5		
67	21	Santana do Riacho III [38,40]	Minas Gerais	–19.16859	–43.6920965	3000	2000	BP	2		
68	8	São Miguel do Tapuio [38]	Piauí	–5.60482	–41.3798965	2500	2000	BP	1		
69	23	Botocudos – Mutum [41]	Espírito Santo	–19.28058	–40.9018158	1663	1954	BC/AD	1		
70	27	Botocudos - Rio Doce [41]	Minas Gerais	–19.64434	–42.4868145	1464	1802	BC/AD	2		
71	28	Botocudos - Cachoeiro de Itapemirim [41]	Espírito Santo	–20.82703	–41.1269521	1668	1954	BC/AD	1		

All primary data sources compiled in this dataset are listed in Table 1. The bibliographical references of the primary data sources, for each archaeological site, are refer in brackets on the column Site Name in Table 1 and in extensive form in the References. The geographical position of all archaeological sites where the data originated from are shown in Fig. 1 and Table 1.

In IsoArcH, data are organized in Excel sheets by the level of the information, from the archaeological site itself to the individual samples from this location. Geographical and bibliographical information of the archaeological sites is compiled. Also, information about the archaeological context of the samples is detailed like chronology, funerary patterns and bioarchaeological attributes like sex, age, stature, and others. For the samples themselves, the description is very detailed with information about the sampled skeletal part, bone level preservation, taphonomy features, etc. In the last sheets all the descriptions and results of the measurements are assembled. The all-compiled data is easily accessible, visualized and downloaded through the platform website. All the variables which appear in the dataset are explained in a detailed way in the IsoArcH platform (https://isoarch.eu/) [1].

We intend to update the Brazilian isotopic database in IsoArcH gradually as the new publications releases. The IsoArcH platform is a dynamic repository and new data can be added without any problem.

## 2 Experimental Design, Materials and Methods

Most of the data compiled here have not been published in peer-review, as they comprise master dissertations, doctoral thesis, and research reports. When published in articles in local journals, they are often limiting the reading by non-Brazilian Portuguese readers. On the other hand, peer-reviewed articles in English are also hard access for Brazilian researchers or Brazilian-Portuguese readers.

Data were carefully curated through a systematic literature review. The platforms and repositories used included Google Scholar, the Brazilian repository of scientific articles Scielo (https://www.scielo.br/), the Brazilian repository of researchers CV *Plataforma Lattes* (https://lattes.cnpq.br/), and each different repository of masters and doctoral dissertations or thesis of each Brazilian university with graduate and postgraduate programs in archaeology. In addition, we conducted personal communication with researchers and university library staff asking for works deposit only in their personal and /or institutional libraries, obtaining manuscripts inaccessible otherwise.

This compilation was carried out by gathering data from 33 publications that reported stable isotopes and/or radiocarbon data for archaeological contexts in Brazil between 1991 and 24 November 2021. Such works consist of:

• Peer-reviewed papers published in English in international scientific journals,
• Peer-reviewed papers published in Portuguese in Brazilian or international scientific journals,
• Non-peer-reviewed manuscripts available in special editions of Brazilian scientific journals,
• Book chapters,
• Research and technical reports in Portuguese, Spanish or English,
• Master dissertations in Archaeology written in Portuguese, and
• Doctoral thesis in Archaeology written in Portuguese, English or other languages.

These works were accessed or obtained through:

• Google Scholar (https://scholar.google.com/),
• A Brazilian repository of scientific articles named Scielo (https://www.scielo.br/),
• The Brazilian repository of researcher's CVs, named *Plataforma Lattes* (https://lattes.cnpq.br/),
• Digital repositories of dissertations and thesis from Brazilian universities with graduate and postgraduate programs in Archaeology,
• Personal communication with universities libraries staff who has publications in their institutional libraries.
• Personal communication with researchers who has publications in their personal libraries.

Samples used to perform isotopic analyses in Brazilian Archaeology are mostly from human skeleton fragments. However, the reported isotopic data are, in most of the cases, not accompanied by a bioarcheological and funerary description of the individuals. Such insufficiency of information limits the accurate archaeological interpretation of the isotopic data.

Unfortunately, some do not include tables with summarized data. Instead, data are reported on graphs or charts, where one could get a glimpse of the available data. We include one work of this type in the references [42], however, we decided not to include it in the presented dataset.

## Ethics Statement

This study does not involve any modern human or animal subject.

## CReDiT Author Statement

**Caroline Borges:** Investigation, Conceptualization, Methodology, Data Curation, Writing– original draft preparation, Reviewing & editing; **Ingrid Chanca**: Conceptualization, Writing – original draft preparation, Reviewing & editing; **Kevin Salesse:** Conceptualization, Data Curation, Visualization, Writing – review & editing

## Declaration of Competing Interest

The authors declare that they have no known competing financial interests or personal relationships which have or could be perceived to have influenced the work reported in this article.

## Data Availability

Stable and radiogenic isotope data from Brazilian bioarchaeological samples: a synthesis (Original data) (IsoArcH).

## References

[1] Salesse K., Fernandes R., de Rochefort X., Brůžek J., Castex D., Dufour E. (2018). IsoArcH.Eu: An open-access and collaborative isotope database for bioarchaeological samples from the graeco-roman world and its margins. J. Archaeol. Sci..

[2] Roosevelt A.C. (1991).

[3] Carvalho L.M.de S. (2020). Aspectos alimentares inferem modos de vida dos povos pretéritos na Serra da Capivara.

[4] De Masi M.A.N. (2001). Pescadores coletores da costa sul do Brasil. Pesquisas: Antropologia.

[5] De Masi M.A.N. (2009). Aplicações de isótopos estáveis de o, c e n em estudos de sazonalidade, mobilidade e dieta de populações pré-históricas no sul do brasil. Revista de Arqueologia.

[6] Oppitz G. (2015).

[7] Plens C.R. (2007).

[8] Eggers S., Parks M., Grupe G., Reinhard K.J. (2011). Paleoamerican diet, migration and morphology in Brazil: Archaeological complexity of the earliest americans. PLoS One.

[9] Milheira R.G., Loponte D.M., García Esponda C., Acosta A., Ulguim P. (2017). The first record of a precolumbian domestic dog (*Canis lupus familiaris*) in Brazil. Int. J. Osteoarchaeol..

[10] Chanca I., Borges C., Colonese A.C., Macario K., Toso A., Fontanals-Coll M., dos Anjos R., Muniz M., Pereira R., Talamo S., Milheira R.G. (2021). Food and diet of the pre-columbian mound builders of the Patos lagoon region in southern Brazil with stable isotope analysis. J. Archaeolog. Sci..

[11] Milheira R.G., Attorre T., Borges C. (2019). Construtores de cerritos na laguna dos Patos, Pontal da Barra, sul do Brasil: Lugar persistente, território e ambiente construído no Holoceno recente. Latin Am. Antiquity.

[12] Bastos M.Q.R. (2009).

[13] M.Q.R. Bastos, S.M.F.M.D. Souza, R.V. Santos, B.A.F. Lima, R.V. Santos, C. Rodrigues-Carvalho. Human mobility on the brazilian coast: an analysis of strontium isotopes in archaeological human remains from Forte Marechal Luz sambaqui. (2011). 10.1590/s0001-37652011000200030 21670891

[14] Bastos M.Q.R. (2014).

[15] Bastos M.Q.R., Lessa A., Rodrigues-Carvalho C., Tykot R.H., Santos R.V. (2014). Análise de isótopos de carbono e nitrogênio: A dieta antes e após a presença de cerâmica no sítio Forte Marechal Luz. Revista do Museu de Arqueologia e Etnologia.

[16] Colonese A.C., Collins M., Lucquin A., Eustace M., Hancock Y., Ponzoni R.A.R., Mora A., Smith C., DeBlasis P., Figuti L., Wesolowski V., Plens C.R., Eggers S., Farias D.S.E., Gledhill A., Craig O.E. (2014). Long-term resilience of Late Holocene coastal subsistence system in Southeastern South America. PLoS One.

[17] Klokler D.M. (2008).

[18] Klokler D.M., Gaspar M.D., Scheel-Ybert R. (2018). Why clam? Why clams? Shell mound construction in southern Brazil. J. Archaeol. Sci..

[19] Pezo-Lanfranco L., DeBlasis P., Eggers S. (2018). Weaning process and subadult diets in a monumental brazilian shellmound. J. Archaeol. Sci..

[20] Pezo-Lanfranco L., Eggers S., Petronilho C., Toso A., Bandeira D.da R., Tersch M.von, Santos A.M.P.D.dos, Costa B.R.da, Meyer R., Colonese A.C. (2018). Middle holocene plant cultivation on the atlantic forest coast of Brazil?. R. Soc. Open Sci..

[21] Afonso M.C., DeBlasis P.A.D. (1994). Aspectos da formação de um grande sambaqui: Alguns indicadores em Espinheiros II, Joinville. Revista Do Museu De Arqueologia E Etnologia.

[22] Scheel-Ybert R. (2012).

[23] Bianchini G.F. (2015).

[24] Hermenegildo T. (2009).

[25] Strauss A. (2016).

[26] Hermenegildo T., O’Connell T.C., Guapindaia V.L.C., Neves E.G. (2017). New evidence for subsistence strategies of late pre-colonial societies of the mouth of the amazon based on carbon and nitrogen isotopic data. Quat Int..

[27] Bastos M.Q.R., Mendonça de Souza S.M.F., Santos R.V., Cook D.C., Rodrigues-Carvalho C. (2011). Da Africa ao cemitério dos pretos novos, Rio de Janeiro: Um estudo sobre as origens de escravos a partir da análise de isótopos de estrôncio no esmalte dentário. Revista de Arqueologia.

[28] Bastos M.Q.R., Santos R.V., Souza S.M.F.M.D.de, Rodrigues-Carvalho C., Tykot R.H., Cook D.C., Santos R.V. (2016). Isotopic study of geographic origins and diet of enslaved africans buried in two brazilian cemeteries. J. Archaeolog. Sci..

[29] Bandeira A.M. (2018). Os sambaquis na Ilha de São Luís – MA: Processo de formação, cultura material cerâmica e cronologia. Revista Memorare.

[30] Colonese A.C., Winter R., Brandi R., Fossile T., Fernandes R., Soncin S., McGrath K. (2020). Matthew Von Tersch, Arkley Marques Bandeira. Stable isotope evidence for dietary diversification in the pre-columbian Amazon. Sci. Rep..

[31] Bastos M.Q.R., Solari A., da Silva S.F.S.M., Martin G. (2019). Estudo preliminar de dieta à partir de isótopos em grupos caçadores-coletores do agreste pernambucano (Holoceno recente - Nordeste do Brasil). FUMDHAMentos.

[32] Solari A., Alves-Pereira A., Sá Espinola Gabriela Martin C., Costa I.P.da, Silva S.F.S.M.da (2016). Escavações arqueológicas no abrigo Pedra do Cachorro. Buíque, PE. Clio Arqueológica..

[33] Solari A., Silva S.F.S.M.da, Mello S.di (2015). Estudo de caso sobre indicadores bioarqueológicos de práticas mortuárias complexas em esqueleto humano coletado no abrigo Pedra do Cachorro. Buíque, PE. Clio Arqueológica..

[34] Solari A., Martin G., da Silva S.F.S.M. (2018). Estudos em bioarqueologia e arqueotanatologia no sítio Pedra do Cachorro, Buíque, PE. Caracterização do sepultamento 3 (3.560 ± 30 ap). Clio Arqueológica.

[35] Bernardo D.V., Neves W.A. (2009). Diversidade morfocraniana dos remanescentes ósseos humanos da Serra da Capivara: Implicações para a origem do homem americano. FUMDHAMentos.

[36] Farias Filho B.B., Soares Meneses Lage M.C. (2012). Laiane De Moura Fontes, Lívia Martins Dos Santos, Luís Carlos Duarte Cavalcante, José Domingos Fabris, José Albertino Bendassolli. Reconstituição da paleodieta de populações humanas de sítios arqueológicos do Parque Nacional Serra da Capivara. Revista de Arqueología Americana.

[37] Guidón N., Pessis A.M., Martin G. (2009). Pesquisas arqueológicas na região do Parque Nacional Serra da Capivara e seu entorno (Piauí –1998 –2008). FUMDHAMentos.

[38] Lima J.O.G.de (2012). Alguns aspectos da dieta de humanos pré-históricos brasileiros. Revista de Arqueología Americana.

[39] Rocha R.L. (2011). Descrição preliminar do sítio arqueológico caixa d’água e de seus remanescentes ósseos humanos (Buritizeiro, Minas Gerais 6.000 BP). Revista do Museu de Arqueologia e Etnologia Suplemento.

[40] Prous A., Malta I.M. (1991). Santana do Riacho - Tomo I. Arquivos do Museu de História Natural da Universidade Federal de Minas Gerais.

[41] Malaspinas A.S., Lao O., Schroeder H., Rasmussen M., Raghavan M., Moltke I., Campos P.F., Sagredo F.S., Rasmussen S., Gonçalves V.F., Albrechtsen A., Allentoft M.E., Johnson P.L., Li M., Reis S., Bernardo D.V., DeGiorgio M., Duggan A.T., Bastos M., Wang Y., Stenderup J., Moreno-Mayar J.V., Brunak S., Sicheritz-Ponten T., Hodges E., Hannon G..J., Orlando L., Price T.D., Jensen J.D., Nielsen R., Heinemeier J., Olsen J., Rodrigues-Carvalho C., Lahr M.M., Neves W.A., Kayser M., Higham T., Stoneking M., Pena S.D., Willerslev E. (2014). Two ancient human genomes reveal polynesian ancestry among the indigenous botocudos of Brazil. Curr. Biol..

[42] Calippo F. (2010).

